# Exploratory screening for Fabry’s disease in young adults with cerebrovascular disorders in northern Sardinia

**DOI:** 10.1186/s12883-015-0513-z

**Published:** 2015-12-12

**Authors:** Laura Fancellu, Walter Borsini, Ilaria Romani, Angelo Pirisi, Giovanni Andrea Deiana, Elia Sechi, Pietro Emiliano Doneddu, Anna Laura Rassu, Rita Demurtas, Anna Scarabotto, Pamela Cassini, Eloisa Arbustini, GianPietro Sechi

**Affiliations:** Department of Clinical and Experimental Medicine, University of Sassari, Viale S. Pietro, 10, 07100 Sassari, Italy; Department of Neuroscience, Psychology, Drug Research and Child Health, University of Florence, Florence, Italy; Centre for Inherited Cardiovascular Diseases, IRCCS Foundation, Policlinico San Matteo, University Hospital, Pavia, Italy

**Keywords:** Fabry’s disease, Stroke, Cerebrovascular, Screening, Prevalence

## Abstract

**Background:**

The etiologic determinants of stroke in young adults remain a diagnostic challenge in up to one-fourth of cases. Increasing evidences led to consider Fabry’s disease (FD) as a possible cause to check up. We aimed at evaluating the prevalence of unrecognized FD in a cohort of patients with juvenile stroke in northern Sardinia.

**Methods:**

For this study, we enrolled 178 patients consecutively admitted to our Neurological Ward for ischemic stroke, transient ischemic attack, intracerebral haemorrhage, neuroradiological evidence of silent infarcts, or white matter lesions possibly related to cerebral vasculopathy at brain MRI, and cerebral venous thrombosis. The qualifying events have to occur between 18 and 55 years of age.

**Results:**

We identified two patients with an α-galactosidase A gene variant, with a prevalence of 0.9 %. According to recent diagnostic criteria, one patient, included for the occurrence of multiple white matter lesions at brain MRI, had a diagnosis of definite FD, the other, included for ischemic stroke, had a diagnosis of uncertain FD.

**Conclusions:**

Our study places in a middle position among studies that found a prevalence of FD up to 4 % and others that did not find any FD patients. Our findings confirm that FD should be considered in the differential diagnosis of patients with juvenile stroke, particularly those with a personal or familial history positive for cerebrovascular events, or evidence of combined cardiologic and/or renal impairment. All types of cerebrovascular disorders should be screened for FD, including patients with white matter lesions possibly related to cerebral vasculopathy at brain MRI.

## Background

Stroke is the first leading cause of disability in adults and the fourth cause of death [1, 2]. In Italy, and other European countries, there are over 200.000 new cases of cerebrovascular disorders (CDs) annually, with the incidence of stroke in young adults peaking at a rate of ten cases per 100.000 inhabitants [3]. CDs in young adults are etiologically heterogeneous and the main clinical challenge in their management remains the identification of the causes. These may be the result of shared environmental and genetic factors, leaning toward the hereditary ones in young adults [4]. Single gene diseases that may present with stroke or transient ischemic attack (TIA), include Fabry’s disease (FD) [5], a rare X-linked inborn error of glycosphingolipids metabolism, caused by mutations in the alpha-galactosidase A (GLA) gene, resulting in the reduced production of the enzyme *α*-galactosidase A. In FD, the enzymatic deficiency leads to lysosomal accumulation of neutral glycosphingolipids in all tissues, particularly in vascular endothelial cells [6]. This is consistent with the natural history of FD that frequently includes the occurrence of different CDs, such as TIAs and stroke, even at very young age in both genders, and the appearance of white matter lesions (WMLs) on brain MRI due to cerebral vasculopathy [6–9]. Since patients with definite FD may be safely treated with a specific enzyme replacement therapy (ERT) with benefit, a timely diagnosis has therapeutic and prognostic implications [8, 10].

Recently, numerous studies investigating FD prevalence in particular populations at risk, such as patients with juvenile stroke, cryptogenic renal and/or cardiac disturbances, have been undertaken [11]. In particular, in the first screening for FD, in patients with juvenile stroke in Germany, a high number of FD patients (about 4 %) have been detected [12]. However, a subsequent Belgian study, conducted on a smaller sample of patients with stroke of unknown origin, was unable to identify any FD patient [13]. Considering these findings, we conducted the present, exploratory study to investigate the prevalence of FD in patients with juvenile stroke in northern Sardinia, a Mediterranean island, with up to now unrecognized FD. Judging precedent inclusion criteria too restrictive, with respect to the CDs qualifying events (QEs), we considered not only cryptogenic ischemic stroke but also the other classes of CDs included in the TOAST classification (Trials of ORG 10172 in Acute Stroke Syndromes) [14, 15] Moreover, we included patients with WMLs at brain MRI, possibly related to cerebral vasculopathy.

## Methods

### Design of the study

The Exploratory Fabry Cerebrovascular disease Screening is the first study designed for assessing the occurrence of FD in Sardinia. We prospectively analyzed patients with CDs admitted to our neurological ward, at Sassari University, in north Sardinia, which allows access to about 100 patients with CDs per year. Among these patients, approximately 20 % are people aged between 18 and 55 years of age. People admitted to our neurological ward come mainly from two north Sardinia areas, which in 2014 had about 480.000 inhabitants and 260.000 people aged between 18 and 55 years of age (2011 population census, ISTAT findings). Inclusion criteria were the following: age between 18 and 55 years of age at the time of the QE, and one of the following QEs, ischemic stroke (IS), TIA, intracerebral haemorrhage (ICH), WMLs at brain MRI possibly due to cerebral vasculopathy, and cerebral venous thrombosis (CVT). We considered the definition of stroke devised by the World Health Organization in the 1970s, “neurological deficit of cerebrovascular cause that persists beyond 24 h” [16]. Considering the multi-tissues involvement in FD patients, with regard to ischemic stroke we chose to consider all the other classes of CDs included in the TOAST classification [14, 15]. Moreover, we included patients with non-traumatic ICH, defined as every spontaneous bleeding into the brain [17]; patients with TIA, defined as an episode with stroke-like symptoms lasting no more than 24 h, [18] and patients with WMLs at brain MRI, even in absence of overt clinical manifestations when possibly due to cerebral vasculopathy [19–21]. CVT was defined as a thrombosis in one or more of cerebral veins.

A diagnosis of definite or uncertain FD was made on the basis of characteristic phenotypic or biochemical features of patients, according to recent diagnostic criteria for FD in adults [22, 23].

Our primary objective was to estimate the prevalence of positive DNA mutations or potentially pathogenic single nucleotide polymorphisms in the GLA gene in an unselected, consecutive group of patients with one or more of the chosen QEs. Our secondary objective was to assess, in each patient, the clinical presentation also in relation to others associated risk factors for CDs, and to point out the specific category of the QE, with special attention to patients with FD, in order to identify possible clues useful in clinical practice.

### Participants and informed consent

All participants enrolled in the study provided written informed consent for enzymatic and genetic testing, and for the publication of individual clinical details. A detailed personal and familiar history, with particular attention to cerebrovascular risk factors and the occurrence of signs and symptoms indicative of FD were noticed. The study protocol was approved by the Human Ethics Review Committees of Sassari and Pavia (Prot. N° P-20070030590).

### Biochemical studies and genetic analysis

A blood sample on Heparin and on EDTA was drawn from all patients, and sent to the Molecular Genetics Laboratory of the Institute of Recovery and Care of Scientific Character (IRCCS) Foundation “S. Matteo”, Pavia. Our patients were also integrated into the polycentric Italian study GIMAF (Interdisciplinary Group for Anderson Fabry Disease). Enzyme alpha-galactosidase A activity was assessed in plasma and leucocytes by spectrofluorimetry, considering normal α-galactosidase A level higher than 2 nmol/mL/h in plasma or 20 nmol/mg prot/h in leucocytes. DNA analysis was done for each of the seven exons of GLA gene, amplified by polymerase chain reaction (PCR). Each PCR product was analyzed by denaturing high-performance liquid chromatography (dHPLC), with a sensibility of 66–98 %, searching for heteroduplex and was examined amplicons with heteroduplex profile by automated DNA sequencing. Concentration of lyso-Gb3 in plasma was measured by tandem mass spectrometry in patients with positive DNA mutations or potentially pathogenic single nucleotide polymorphisms in the GLA gene, and in their relatives.

We used the centralized database of the GIMAF study and a database on our website, including data on clinical variables.

## Results

We identified 187 eligible patients between April 2008 and April 2012. Nine patients were excluded: three patients because they did not give informed consent, the others 6 because they did not fulfill the requirements for definite cerebrovascular events. We included 178 patients (105 women, and 73 men). The average age at recruitment was 48.4+/−22.8 years of age, while the mean age at onset of CDs symptoms was 43.3 years of age (range 20–55 years). The onset of the QE for patients with WMLs refers to the first documentation of the WMLs at brain MRI. None of the patients had previous personal or familiar diagnosis of FD.

Two patients tested positive at the genetic GLA tests: one man aged 41, included for WMLs, who showed a known GLA mutation (R227Q), and severe cardiac and renal involvement, and a woman aged 52, included for recurrent, cryptogenic ischemic stroke, who carried the D313Y mutation of GLA gene and presented with combined cardiac and renal involvement. (Table 1) Moreover, the man had shown acroparesthesias since childhood together with frequent pain crisis, which mainly occurred while doing physical activity and during fever. Also, at brain MRI, he showed the pulvinar sign in T1 sequence, and dolichoectasia of vertebrobasilar artery [24]. The woman, instead, had a first occurrence of stroke at the age of 48, with the brain MRI showing multiple WMLs. Also, she had hypertrophic cardiomyopathy and renal damage with proteinuria and increased serum creatinine. In the man with the known GLA mutation (R227Q), alpha-galactosidase A activity was pathologically decreased to 0.5 μmol/l/h (reference, ≥ 2 μmol/l/h) and the concentration of lyso-Gb3 in plasma was increased to 33.3 ng/ml (reference, ≤1.8 ng/ml). In the woman carrying the D313Y variant, instead, alpha-galactosidase A activity was decreased in plasma, while the concentration of lyso-Gb3 was within normal limits.

**Table 1:** Demographics and risk factors in patients and probands

Demographics	Total number	FD: R227Q	D313Y
Patients	178	1	1
Men	73 (40,7 %)	1	0
Age at recruitment	48,4^a^	41	52
Age at onset (symptomatic)	43,3^a^		48
Age at onset (WMLs)	43,5^a^	41	
Risk factors			
Hypertension	106 (59,5 %)	1	1
Diabetes	11 (6,1 %)	0	0
Atrial fibrillation	3 (1,7 %)	0	0
Dyslipidemia	83 (46,6 %)	0	1
Smoking	40 (22,4 %)	0	0
Drinking habit	13 (7,3 %)	0	0
Oral contraception	5 (4,7 %)	NA	0
Hyperhomocysteine	22 (12,3 %)	1	1
Overweight/obesity	68 (38,2 %)	0	1
Fabry feature			
Acroparesthesia	13 (7,3 %)	1	0
Angiokeratoma	0	0	0
Cornea verticillata	1 (0,5 %)	0	0
CKD/Proteinuria	22 (12,3 %)	0	1
Cardiac involvement	32 (17,97 %)	1	1

*Abbreviations*: *FD* Fabry’s disease, *NA* not applicable, ^a^median *NB* cornea verticillata was associated with amiodarone

Considering the distribution of QEs: 107 patients presented IS (59 women and 48 men), 30 TIA, 30 WMLs, eight ICH and three women CVT. Table 2 shows the etiopathogenetic TOAST classes we found. Patients with WMLs came to our attention because of different neurological disturbances: 11 of them showed gait disturbances, ten headache, six vertigo and dizziness, one an epileptic seizure, one an acute confusion state, one syncopal episodes.

**Table 2:** Fabry diagnosis in the different cerebrovascular diseases

	Our study	Rolfs 2005	Brouns 2007	Brouns 2010	Wozniak 2010	Baptista 2010	Sarikaya 2012	Marquardt 2012	Dubuc 2012	Rolfs 2013
Sample size	178	721	103	1000	558	493	150	1046	100	5023^g^
IS	107 (60,1 %)	721 (100 %)	57 (55,3 %)	573 (57.3 %)	1°stroke 558(100 %)	364(73,8 %)	135(90 %)	572(54,6 %) 4^a^	100(100 %)	3291(65,51 %)
Atherothrombotic	11(10,3 %)	0	0	143 (25,1 %)	NR	55(15,1 %)	0	NR	/	613(18,6 %)
Cardioembolic	11(10,3 %)	0	0	125 (21,9 %)	NR	80 (21,9 %)	0	NR	/	549 (16,7 %)
						1^d^; 1^a^				
Lacunar	17(15,8 %)	0	0	99 (17,4 %)	NR	98 (26,9 %)	0	NR	13(13 %)	443 (13,5 %)
						1^d^; 1^a^				
Other determined	6(5,6 %)	0	0	69 (12,1 %)	NR	27(7,4 %)	0	NR	/	585 (17,8 %)
				2^c^		1^d^				
Undetermined	62(57,9 %)	721	57(100 %)	134 (23,5 %)	154 (28 %\)	104(28,5 %)	135 (100 %)	NR	/	1100 (33,4 %)
	1^a^	32^f^		1^e^		1^d^; 3^a^				
TIA	30(16,8 %)	NR	28(27,1 %)	220(22 %)	/	NR	15 (10 %)	474(45,3 %)	/	1071 (22,3 %)
				3^a^				1^a^		
ICH	8(4,5 %)	NR	18(17,4 %)	49 (4,9 %)	/	115(23,3 %)	/	/	/	ICH 271(5,6 %)
						1^d^; 1^a^				CVT/SAH 68 (1,4 %)
CVT	3(1,6 %)	/	/	/	/	14(2,8 %)	/	/	/	
						1^d^				
WMLs	30(16,8 %)	/	/	153(15,3 %)	/	76(15,4 %)	/	/	/	/
	1^b^			2^a^		1^d^; 2^a^				

*Abbreviations*: *IS* ischemic stroke, *TIA* transient ischemic attack, *ICH* intracerebral haemorrhage, *SAH* subarachnoid haemorrhage, *CVT* cerebral venous thrombosis, *WMLs* white matter lesions, *NR* not reported, ^a^D313Y; ^b^R227Q; ^c^A143T; ^d^R118C; ^e^S126G; ^f^mutations NR; ^g^mutations found [R118C, V315I, S126G (3×), A143T (4×), D83N, L415F, S102L, E418G]; /, not available

With regard to cardiovascular risk factors, we found a high percentage of hypertension, (59.5 %) and hyperlipidemia (46.6 %), overweight (47.6 %), smoking (24.2 %) and hyperhomocysteinemia (14 %).

### Of note, our FD patients were included in the study on average 5 years after the qualifying event. During this time frame, in these patients renal damage progressed with increase of proteinuria and serum creatinine

**Fig. 1:**
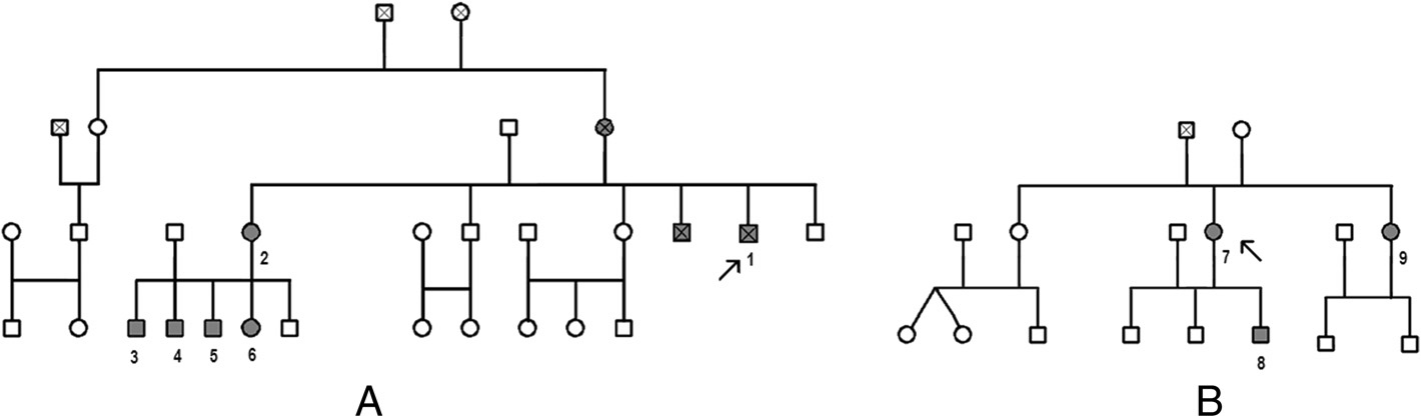
Family trees: Family tree (**a**) and family tree (**b**). Probands are indicated by *arrows*. Patients are numbered as in Table 3

We also analyzed 22 relatives (12 women and ten men) of the two probands. In the first family group, out of 16 relatives examined, two women and three men resulted affected. In the second family, out of the six relatives studied, two resulted positive to FD at genetic tests, one man and one woman. The clinical features of the two families are shown in Table 3 and the family trees are presented in Fig. 1.

**Table 3:** Clinical features and enzyme analyses of the Fabry patients

Patients	Gender	Age at	Manifestations				α-galactosidase A	Lyso-Gb3
							(RV: ≥2 μmol/l/h)	(RV: ≤1.8 ng/ml)
		Diagnosis	Acroparesthesia	Cerebrovascular	Cardiac	Renal		
P 1^a^	M	41	+	WMLs, pulvinar sign	Left ventricular hypertrophy	CKD	0.5	33.3
2^a^	F	51	+	-	Silent acute coronary syndrome	-	8.1	5,6
3^a^	M	31	+	-	-	Proteinuria	0.66	31.1
4^a^	M	29	+	-	Atrial fibrillation	-	2	/
5^a^	M	23	+	-	-	-	2	51
6^a^	F	18	+	-	-	-	6.6	4.7
P 7^b^	F	51	-	Recurrent stroke and WMLs	Left ventricular hypertrophy	CKD	15.4	2.5
8^b^	M	21	-	-	-	-	2	/
9^b^	F	49	-	-	-	-	5.3	1.3

*Abbreviations*: *P* proband; ^a^R227Q, ^b^D313Y, + yes, − no, *RV* reference values, /,not available

## Discussion

Several studies indicate that patients with FD have a 20-fold increased risk of ischemic stroke and TIA compared to general population [24–26]. Multiple CDs arising in young and middle-aged adults have been described in FD. Stroke is the most frequent, and can develop either as a consequence of cardiac embolism, or due to primary cerebral vasculopathy. In patients with FD, the cerebral vasculopathy is related to damage in small and large blood vessels, mainly at level of vertebro-basilar circulation [24, 26]. In a cohort study of 2446 patients from the Fabry Registry, 138 patients experienced stroke (about, 5.6 %). Half of these patients experienced their first stroke before being diagnosed with FD, and 70 % before any renal or cardiac disturbance [9]. Thus, it is important to search for FD in patients with early onset CDs. In literature, there is a wide variability, from 4 to 0 %, in the reported prevalence of FD in patients with cryptogenic stroke (Table 2) [12, 13, 27–32]. This is probably due to different methodological approaches, in particular the choice of different QEs, and the size of the sample analyzed. In our study, we chose to include several kinds of CDs, as previously detailed. In other studies, [33, 34] a wide range of different methods were used, in particular as regards the choice of QEs. The most recent study on prevalence of FD in young adults with CDs comes from the multinational European study Stroke in Young Fabry Patients (Sifap) [35]. This study, in a cohort of 5023 patients, aged between 18 and 55 years, found that the prevalence of definite FD was 0.5 %, with an adjunctive 0,4 % prevalence for probable FD, including in this definition patients carrying the D313Y variant [36]. In our study, according to recent diagnostic criteria in adults [22, 23], comprehensively, we found a 0,9 % prevalence of FD in young people with CDs, namely, 0,45 % of definite FD, plus 0,45 % of uncertain FD, including the patient with the D313Y variant genotype.

This is the first study that documents the occurrence of FD in neurologic patients in Sardinia island. Our result places in a middle position between the 4 % prevalence firstly reported in the study by Rolfs [12] (probably, overestimated), and the studies that found no FD patients in their clinical records (probably, underestimated) [13, 28]. In our study, two patients with a GLA gene variant were identified: a man, included for the occurrence of WMLs possibly due to cerebral vasculopathy at brain MRI, who showed the known GLA mutation (Arg227Gln), associated with a classical phenotype of FD. This patient had a profound decrease of alpha-galactosidase A activity in plasma and leukocytes, and an increased concentration of lyso-Gb3 in plasma; and a woman, included for recurrent ischemic stroke, who carried the D313Y GLA gene variant, commonly defined as polymorphism and reportedly associated with lower alpha-galactosidase A level in plasma and normal GLA enzyme activity in leukocytes, causing the so called “pseudodeficiency” [36]. In this woman, the concentration of lyso-Gb3 in plasma was within normal limits. Recently, this mutation, that was formerly reported as nonpathogenic, has been associated with multifocal WMLs at brain MRI and exclusive neurologic manifestations [37]. Of note, mono-organic manifestations have been frequently described with prominent cardiac or renal manifestations in FD [37]. Moreover, several studies reported an association of D313Y with other typical FD manifestations, such as stroke, renal failure, peripheral neuropathty, or hypertrophic cardiomyopathy [27, 33, 38]. In particular, a recent prospective study including 625 patients with cerebral ischemia, aged between 18 and 55 years, reported that this gene variant was associated with cryptogenic stroke [33]. Notably, the occurrence of a high proportion of FD in recurrent stroke is suggested by our findings and it has also been reported in the German and Belgian studies (Table 2) [12, 13, 34, 35].

Moreover, an analysis of studies in literature indicates that frequent etiologies of ischemic stroke in patients with FD, according to the TOAST classification, include the following: cryptogenic, cardioembolic, other determined and lacunar [12, 13, 27–35]. Interestingly, in literature, FD diagnosis has been never reported in patients with atherothrombotic strokes. FD diagnosis, instead, has also been reported in patients with TIA, ICH, occurrence of WMLs at brain MRI and CVT. We emphasize the potential clinical relevance of searching FD in patients with only WMLs. This is related to the continuing search for clinical or paraclinical markers useful for an early diagnosis in FD patients, and consequently a timely ERT in definite FD patients [10, 22, 23], with the possibility of delaying the natural course of the disease.

Sixteen per cent of our patients were included for WMLs at brain MRI, and we found our first patient in this group. WMLs are quite common in the elderly general population, but they are distinctly uncommon among the general population under 55 years of age [39]. A high prevalence of WMLs at MRI and silent infarcts were found in several studies in patients with cerebrovascular and cardiovascular disorders [39–41]. They have been found in patients under 49 years of age, in 8 % in the Framingham Offspring Study and the Helsinki Young Stroke Registry [41, 42]. Putaala et al. reported a 7 % prevalence of WMLs in a similar range of age [39]. Moreover, several studies reported a high prevalence of WMLs in FD patients, reaching 100 % after 55 years of age [42, 43]. Taken together, these findings indicate the need for a careful search for FD in this population. Further details on the distribution of FD diagnosis in relation to the inclusion criteria of our patients are reported in Table 2. Moreover, analyzing cardiovascular risk factors in our study population, we found a high percentage of hypertension, hyperlipidemia, overweight/obesity and hyperhomocysteinemia, in line with other similar screening studies.

## Conclusions

Our data indicate the necessity to evaluate FD prevalence in patients with cerebrovascular disease in young age in the entire Sardinia island. In particular, the diagnosis of FD should be considered in patients with cryptogenic stroke and in patients with WMLs at brain MRI possibly related to cerebral vasculopathy, especially if other FD related symptoms are present. Attention should also be paid to recurrent stroke, which may hide a large proportion of FD patients. In patients at risk, a complete and detailed personal and family history should be obtained, together with an accurate search for clinical features indicative of FD at physical examination. Since FD is now a potentially treatable clinical condition, an early diagnosis may have relevant prognostic implications for patients and their relatives. Neurologists may have a prominent role in an early identification of this pathology.

## Abbreviations

CDs: cerebrovascular dieases
CKD: chronic kidney disease
CVT: cerebral venous thrombosis
ERT: enzyme replacement therapy
FD: Fabry’s disease
ICH: intracerebral haemorrhage
QEs: qualifying events
TIA: transient ischemic attack
WMLs: white matter lesions
